# Mechanisms of resistance to antibody-drug conjugates in cancer therapy: molecular basis and therapeutic strategies

**DOI:** 10.20517/cdr.2025.148

**Published:** 2025-11-28

**Authors:** Yue Hao, Zhengbo Song

**Affiliations:** ^1^Jinling Hospital, Affiliated Hospital of Medical School, Nanjing University, Nanjing 210002, Jiangsu, China.; ^2^Department of Clinical Trial, Zhejiang Cancer Hospital, Hangzhou 310022, Zhejiang, China.

**Keywords:** Antibody-drug conjugates, resistance, mechanism, challenges, future directions

## Abstract

Antibody-drug conjugates (ADCs) have emerged as a transformative class in oncology, integrating the target specificity of monoclonal antibodies with the potent cytotoxicity of small-molecule payloads. By harnessing tumor-specific antigen recognition, ADCs enable the selective delivery of chemotherapeutic agents, thereby enhancing therapeutic efficacy while reducing systemic toxicity. Their clinical success across both hematologic malignancies and solid tumors underscores their potential to redefine targeted cancer therapy. However, the clinical durability of ADCs is increasingly undermined by the emergence of diverse resistance mechanisms that diminish their antitumor activity. These mechanisms encompass the entire drug delivery cascade - from reduced or heterogeneous antigen expression and impaired internalization to defective lysosomal trafficking, enhanced drug efflux, and payload detoxification. In addition, adaptive reprogramming of oncogenic signaling pathways and tumor microenvironmental factors can further attenuate ADC cytotoxicity and promote tumor persistence. A comprehensive understanding of the molecular and cellular bases of ADC resistance is essential for sustaining their therapeutic impact. Advances in linker chemistry, innovative payload design, and the development of bispecific or immune-modulating ADCs offer promising strategies to overcome these challenges. Concurrently, the integration of biomarker-driven patient selection and rational combination regimens is poised to enhance treatment precision and delay resistance. Continued mechanistic and translational research will be pivotal to fully realizing the potential of next-generation ADCs in precision oncology.

## INTRODUCTION

With the growing demand for precision medicine, targeted therapies that inhibit tumor growth have significantly improved treatment accuracy and efficacy. Several targeted therapeutic strategies have already been widely applied in clinical practice, particularly in patients harboring oncogenic driver mutations^[1]^. Over the past few decades, the antitumor efficacy of chemotherapy has been well established across various malignancies. However, its widespread application has been limited by high rates of drug resistance and severe adverse effects^[2]^. To enhance the efficiency of chemotherapy, alleviate acquired resistance, and minimize systemic toxicity, a new therapeutic modality - antibody-drug conjugates (ADCs) - has been developed^[3]^. ADCs consist of three key components: a monoclonal antibody, a cytotoxic payload, and a chemical linker connecting the two. This rational design enables the integration of precise tumor targeting with potent cytotoxic activity, thereby achieving selective tumor cell killing while sparing normal tissues^[4]^. Several ADCs have been approved by the U.S. Food and Drug Administration (FDA)^[3]^. For example, datopotamab deruxtecan has been approved for the treatment of breast cancer, demonstrating superior efficacy compared with conventional chemotherapy^[5]^. Moreover, more than 80 ADCs have reached clinical use, and nearly 600 others are currently undergoing clinical evaluation^[6,7]^.

**Figure 1:**
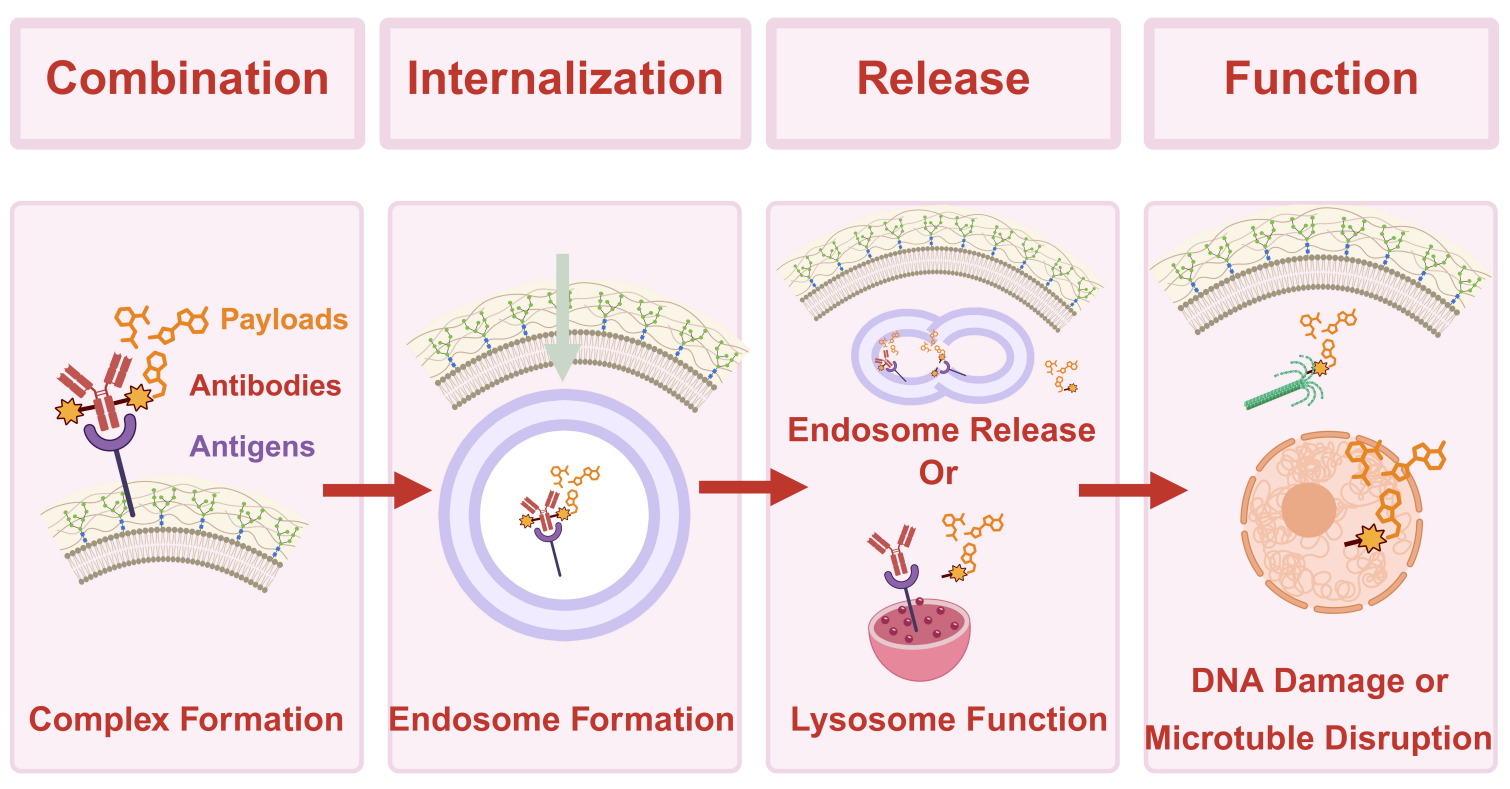
Mechanism of action of ADC drugs in the whole process. The antibody component of an ADC specifically recognizes and binds to a target antigen on the cell membrane. Following binding, the complex is internalized via endocytosis. Once inside the cell, the ADC is transported to lysosomes, where it undergoes degradation. The linker connecting the antibody to the cytotoxic payload is cleaved by proteolytic enzymes, releasing the cytotoxic drug into the cytoplasm. Subsequently, the released drug disrupts the cell cycle, leading to tumor cell death. Created in BioRender. Hao, Y. (2025) https://BioRender.com/4xpt5v5. ADC: Antibody-drug conjugate.

The pharmacologic precision of ADCs is attributed to their unique structure, which dictates their functional mechanism *in vivo*. The antibody component recognizes specific antigens on the surface of target cells and mediates internalization through endocytosis^[8]^. Within the acidic environment of the lysosome, the ADC is degraded, and the linker between the antibody and the cytotoxic payload is cleaved by proteolytic enzymes^[9]^. Subsequently, the released cytotoxic agent enters the cytoplasm, disrupts the cell cycle, and induces apoptosis. The overall mechanism of ADC action is illustrated in Figure 1. Given their innovative design and precise tumor-killing capability, ADCs hold broad therapeutic potential. Several ADCs are expected to achieve further clinical translation and therapeutic enhancement in the near future. Nevertheless, as their clinical use expands, resistance to ADCs has increasingly emerged as a significant challenge. Reports of acquired resistance are accumulating, underscoring the urgent need for a systematic analysis of the underlying mechanisms and clinical challenges^[10]^. The present study reviews the potential mechanisms of ADC resistance and discusses their implications for clinical therapy.

## MECHANISMS OF RESISTANCE TO ADCS

**Figure 2:**
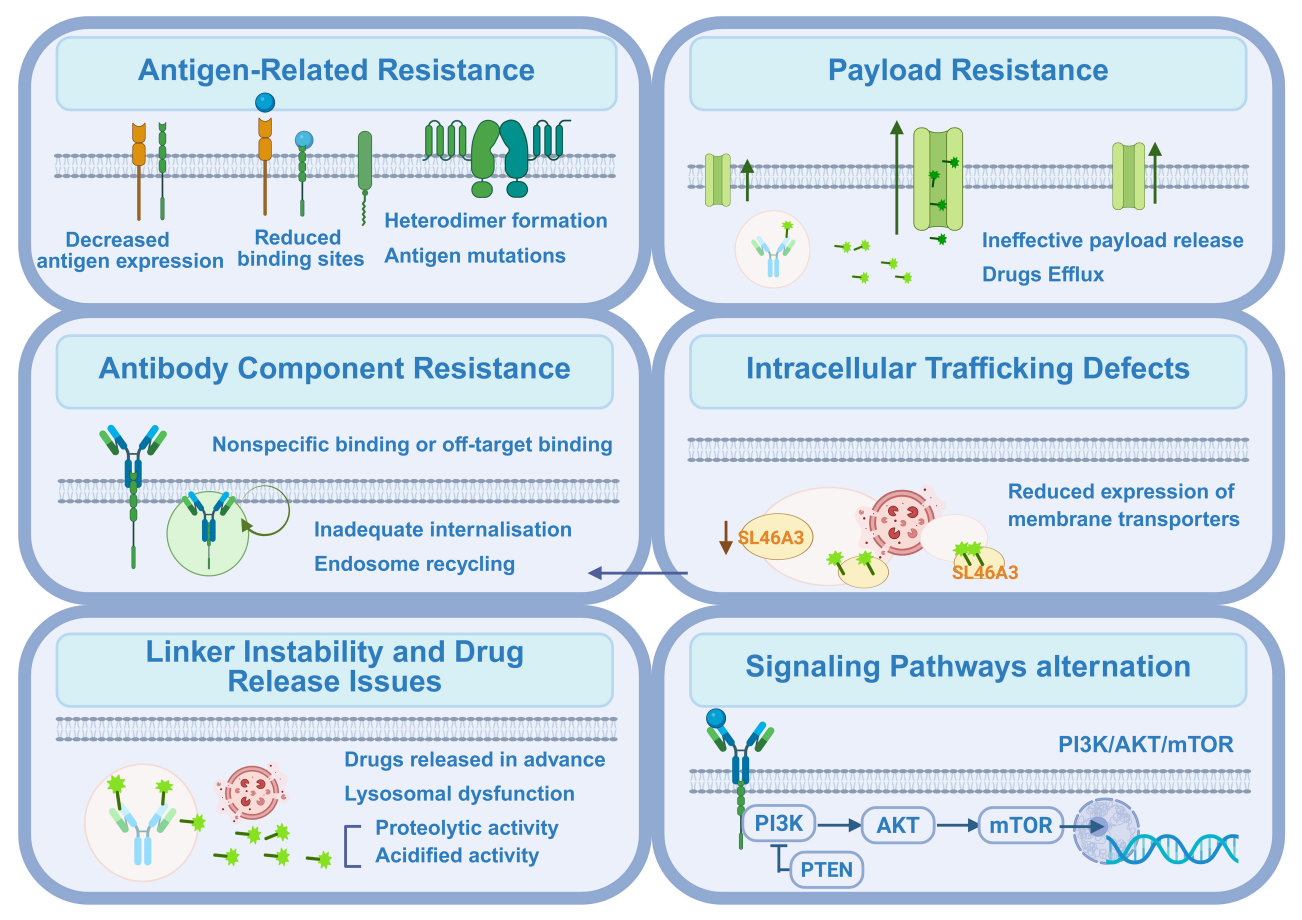
Main mechanisms of resistance to ADC drugs. Resistance to ADCs arises through multiple mechanisms that impair their efficacy at various stages. The main mechanisms include the antigen-related resistance, payload resistance, antibody component resistance, intracellular trafficking defects, linker instability and drug release issues, as well as alterations in signaling pathways. Created in BioRender. Hao, Y. (2025) https://BioRender.com/2b91iun. ADC: Antibody-drug conjugate; PI3K: phosphoinositide 3-kinase; AKT: AKT serine/threonine kinase; mTOR: mechanistic target of rapamycin; PTEN: phosphatase and tensin homolog.

Resistance can emerge at multiple stages of ADC activity, including reduced antigen expression, impaired internalization, disrupted intracellular trafficking, lysosomal dysfunction, increased drug efflux, and adaptive alterations in signaling pathways. In addition, tumor heterogeneity and microenvironmental influences further complicate therapeutic outcomes. A deeper understanding of these mechanisms is vital for the rational design of next-generation ADCs, the development of effective combination strategies, and the identification of predictive biomarkers. The following sections will examine these potential resistance mechanisms in detail, with the most common pathways illustrated in Figure 2.

### Antigen-related resistance

Antigen recognition represents the initial step in the functional mechanism of ADCs, determining the specific interaction between an antigen and its corresponding antibody. Prolonged antigen exposure to ADCs can lead to decreased antigen expression levels and fewer available drug-binding sites^[11]^. In addition, structural alterations - such as the loss or mutation of surface antigens - may further diminish ADC binding, resulting in resistance development and impaired internalization of active components.

In breast cancer cell lines, human epidermal growth factor receptor 2 (HER2) downregulation has been observed following exposure to trastuzumab emtansine (T-DM1). Similarly, evidence from multiple myeloma studies indicates that treatment with belantamab mafodotin can induce B-cell maturation antigen (BCMA) loss or escape, which is strongly associated with subsequent treatment failure^[12]^. Comparable downregulation of trophoblast cell surface antigen 2 (TROP-2) has also been detected in triple-negative breast cancer (TNBC) cells exposed to TROP-2-targeted ADCs^[13]^. Interestingly, the reduction in TROP-2 expression also decreased the invasive capacity of TNBC cell lines, suggesting that the malignant potential of cancer cells may be attenuated^[13]^. Some studies have proposed that combining ADCs with other targeted agents may mitigate the effects of antigen downregulation or loss^[14]^. Notably, a growth-inhibitory effect has been observed even in tumors with low TROP-2 expression^[15]^. However, other reports suggest that excessively high antigen loads can paradoxically reduce drug efficacy^[16]^. Similar findings have been reported for ADCs targeting Nectin-4, where changes in antigen expression were associated with diminished therapeutic effects^[17]^. During metastatic progression of urothelial carcinoma, membranous Nectin-4 expression often declines, and this reduction has been linked to resistance to enfortumab vedotin^[18]^.

Caveolin-1 (CAV-1) has been identified as one of the pathways involved in ADC internalization. Studies have demonstrated that CAV-1 overexpression is inversely correlated with HER2 expression on the cell membrane and is associated with reduced colocalization with lysosomes, ultimately leading to decreased sensitivity to T-DM1^[19]^. Preclinical findings further indicate that the colocalization of CAV-1 with T-DM1 corresponds to diminished therapeutic efficacy^[19]^. In contrast, the efficacy of trastuzumab deruxtecan (T-DXd) in HER2-low and HER2-negative breast cancers is not dependent on HER2 binding or ADC internalization. Instead, its antitumor activity is mediated by extracellular proteases within the tumor microenvironment, particularly cathepsin L (CTSL). Irrespective of HER2 expression status, both tumor and stromal compartments of invasive breast cancers exhibit high levels of CTSL, which efficiently cleaves T-DXd’s specialized linker, enabling payload release and exerting cytotoxic effects against HER2-low or HER2-negative tumors^[20]^. Ongoing studies continue to investigate these mechanisms, and the emergence of paradoxical findings has sparked debate among researchers in the field.

On the other hand, tumor heterogeneity in antigen expression can markedly influence the efficacy of ADCs. In a study evaluating T-DM1 in combination with pertuzumab for patients exhibiting HER2 heterogeneity, none achieved the primary endpoint, and the pathologic complete response rate was 0%^[21]^. The findings also revealed that the proportion of HER2 non-amplified cells is a key factor contributing to cellular resistance^[21]^. In addition, the accumulation of truncated extracellular domains of the target antigen has been linked to increased tumor resistance to therapy^[22]^. It is well established that instability in antigen expression levels can compromise the therapeutic effectiveness of ADCs. As antigen recognition constitutes the initial step in ADC function, it plays a pivotal role in the overall mechanism of action. While the specificity and surface density of target antigens are crucial for ADC efficacy, maintaining a relatively stable level of antigen expression throughout the course of treatment is equally essential to ensure sustained therapeutic response.

Furthermore, the interplay between the bystander effect of ADCs and the proportion of antigen-positive cells may significantly influence treatment efficacy. In ADCs with non-cleavable linkers, the bystander effect is often diminished due to limited membrane permeability, which can concurrently reduce overall therapeutic effectiveness^[23]^. Antigen heterogeneity, characterized by variable expression levels across different tumor regions, also contributes to treatment resistance. Some tumor cells may alter the antigens on their membranes or shield them from recognition by secreting mucin or hyaluronan, which serve as physical barriers that impede antibody binding^[7]^. Additionally, antigens may form complexes with other proteins, further promoting drug resistance. For instance, the formation of HER2/human epidermal growth factor receptor 3 (HER3) heterodimers has been implicated in trastuzumab resistance^[24]^. Moreover, HER3 overexpression has been associated with ADC resistance, a condition that can be reversed by HER3 inhibitors in gastric cancer xenograft models^[25]^.

### Antibody component resistance

The interaction between an antibody and its target antigen is a mutually dependent process. Factors such as antigen expression level, intrinsic heterogeneity, antigen shedding, epitope mutations, or other structural alterations of the target antigen can all impair effective antibody-antigen binding. This disruption subsequently interferes with the antigen-mediated internalization pathway upon which ADCs depend. Moreover, nonspecific binding or aberrant intracellular trafficking may misdirect the antibody-antigen complex to non-lysosomal compartments or recycling pathways, thereby hindering efficient payload release.

In addition, a tumor microenvironment that shifts toward an immunosuppressive state may further contribute to drug resistance. Engineering of the fragment crystallizable (Fc) region to reduce Fc gamma receptor (FcγR) binding can also compromise the immunogenic integrity of the Fc domain, consequently weakening complement component 1q (C1q) engagement and attenuating essential effector mechanisms such as the antibody-dependent cellular cytotoxicity (ADCC) and complement-dependent cytotoxicity (CDC)^[26]^.

### Linker instability and drug release issues

The linker is a critical determinant of ADC performance, as it dictates both the timing and location of payload release. Following ADC-antigen binding and internalization, efficient lysosomal processing enables proteolytic enzymes to degrade the conjugate and cleave the linker, thereby liberating the active cytotoxic drug. However, instability in certain linkers can cause premature payload release before the ADC reaches the lysosome. Such off-target release diminishes on-target potency, increases systemic toxicity, and may promote drug resistance by exposing healthy tissues or sublethally dosing tumor cells^[27]^. Consequently, selecting a linker with optimal stability and lysosomal cleavability is essential to balance efficacy and safety.

After target recognition at the cell surface, ADC-antigen complexes are internalized via receptor-mediated endocytosis and sequentially trafficked through early endosomes - where sorting occurs - to late endosomes, which subsequently mature and fuse with lysosomes to enable payload release. Within the acidic, protease-rich lysosomal environment, the antibody is degraded and the linker cleaved, freeing the active payload to exert its cytotoxic effects. This pathway can be disrupted by genetic or molecular alterations that impair internalization or trafficking efficiency - for instance, mutations within the HER family that alter receptor dynamics, or inhibition of HSP90, which destabilizes receptor conformation and endocytic routing - ultimately reducing lysosomal delivery and payload release^[28,29]^.

Thus, robust linker design and intact endocytic-lysosomal trafficking function as interdependent prerequisites for effective ADC activity: the former ensures that payload release occurs precisely in the intended intracellular compartment, while the latter guarantees that the ADC reaches that destination. Any deficiency in either process compromises on-target efficacy and facilitates the development of therapeutic resistance.

### Payload resistance

Overexpression of efflux pumps represents another key mechanism of resistance, wherein the effective payload is expelled from tumor cells before exerting its cytotoxic function. Increased activity of adenosine triphosphate (ATP)-binding cassette (ABC) transporters facilitates the efflux of cytotoxic agents such as monomethyl auristatin E (MMAE), a common ABC transporter substrate^[8]^. Several ADC payloads are recognized as substrates of these transporters, and their upregulation in tumor cells can actively drive drug efflux, thereby diminishing therapeutic efficacy and promoting resistance^[30,31]^. Payload classes including maytansinoids [e.g., emtansine (DM1), soravtansine (DM4)], auristatins [e.g., MMAE, monomethyl auristatin F (MMAF)], and certain topoisomerase I (TOP1) inhibitors [e.g., deruxtecan (DXd), 7-ethyl-10-hydroxycamptothecin (SN-38)] display varying degrees of susceptibility to active efflux, leading to reduced intracellular drug accumulation and attenuated cytotoxic potency^[19,32,33]^. Notably, intact ADCs differ from their unconjugated drugs in efflux susceptibility: the macromolecular conjugate itself is generally not an efflux substrate until lysosomal processing releases membrane-permeable catabolites, which are then efficiently exported by ABC transporters. Thus, linker chemistry, payload hydrophobicity, and release kinetics are critical determinants of how efflux activity ultimately impacts ADC potency.

Alterations in drug targets also contribute to resistance. Following exposure to T-DM1, modifications in the payload target, TOP1, have been observed. Point mutations in TOP1 are thought to reduce the enzyme’s affinity for DNA, thereby weakening the interaction between the payload and the enzyme-DNA complex^[34,35]^. Similarly, mutations affecting tubulin - the target of microtubule-disrupting payloads - can compromise ADC efficacy. Moreover, given that ADC payloads are fundamentally chemotherapeutic agents, they may inherit intrinsic resistance mechanisms associated with conventional chemotherapy^[36]^.

The effective drug-to-antibody ratio (DAR) is another critical factor influencing ADC performance. Site-specific cysteine-conjugated ADCs have demonstrated improved tolerability and enhanced therapeutic outcomes^[37]^. However, the optimal DAR remains undefined, as higher DARs are often associated with accelerated clearance, which can paradoxically reduce efficacy^[38]^. Achieving an optimal balance among conjugation site, conjugation method, and drug loading continues to be an important area of ongoing research.

### Intracellular trafficking defects

During the process of drug endocytosis, some endosomes may undergo recycling, resulting in the rerouting of ADCs away from lysosomes and consequently impairing payload release. Prior to payload liberation, these recycling endosomes can return to the cell membrane, leading to premature clearance of ADCs from the intracellular compartment^[39]^. In addition, the lysosomal membrane plays a crucial role in ADC trafficking and payload processing. For ADCs containing non-cleavable linkers, the lysosomal membrane participates directly in the transport of ADC catabolites. Specific membrane transport proteins, such as solute carrier family 46 member A3 (SLC46A3), facilitate the export of active components from the lysosome to the cytoplasm^[40]^. Reduced expression of these transporter proteins can decrease ADC efficacy and contribute to resistance. Indeed, the role of SLC46A3 loss in conferring resistance to T-DM1 has been demonstrated in the T-DM1–resistant BT-474M1 breast cancer cell line (BT-474M1 is a derivative subline of the human breast cancer cell line BT-474 and is widely employed as a well-characterized model of acquired T-DM1 resistance)^[41]^.

Another potential contributor to ADC resistance arises from limitations in systemic circulation and tissue distribution. Owing to the large molecular size of monoclonal antibodies, their ability to penetrate the blood-brain barrier (BBB) is inherently restricted. Consequently, the effectiveness of ADCs in controlling localized brain lesions remains controversial and varies across studies^[42]^. Notably, T-DXd has demonstrated evidence of central nervous system activity in patient-derived xenograft models of HER2-positive and HER2-low breast cancer brain metastases^[43]^. Preliminary clinical data have also shown efficacy in patients with brain metastases across multiple institutions^[43]^. In contrast, patients with brain metastases treated with T-DM1 did not experience a significant progression-free survival benefit, underscoring the uncertainty surrounding ADC efficacy in treating intracranial disease^[44]^.

Moreover, lysosomal dysfunction represents another mechanism contributing to ADC resistance. Impaired proteolytic degradation and reduced acidification can hinder payload release, thereby diminishing cytotoxic activity^[45]^. Elevated lysosomal pH disrupts the organelle’s degradative capacity, a phenomenon observed in trastuzumab emtansine-resistant cells^[46]^. As lysosomal alkalinization progresses and proteolytic enzyme activity declines, BT-474 breast cancer cells gradually acquire resistance to T-DM1^[41]^.

### Signaling pathways alternation

Multiple alterations in cellular signaling pathways play pivotal roles in modulating ADC function and driving the emergence of drug resistance. These pathways regulate critical biological processes, including drug responsiveness, intracellular trafficking, cell proliferation, apoptosis, and DNA repair. The following section provides a structured overview of the major signaling alterations implicated in ADC resistance, highlighting the interconnections and logical transitions among these mechanisms.

## PI3K PATHWAY ACTIVATION

Previous studies have demonstrated that activation of the phosphoinositide 3-kinase (PI3K) signaling pathway can diminish ADC responsiveness by reducing cytotoxic payload activity and promoting cell survival. In particular, patients harboring phosphatase and tensin homolog (PTEN) loss or phosphatidylinositol-4,5-bisphosphate 3-kinase catalytic subunit alpha (PIK3CA) mutations often exhibit reduced therapeutic efficacy following trastuzumab treatment, largely due to hyperactivation of this pathway^[47]^.

## ANTIGEN EXPRESSION AND WNT/β-CATENIN SIGNALING

Beyond the PI3K pathway, alterations in antigen expression can also modulate downstream signaling cascades. For instance, cell surface expression of TROP-2 has been linked to the activation of signaling pathways that enhance cellular proliferation and invasiveness, thereby contributing to ADC resistance^[48]^. Similarly, activation of the wingless/integrated (Wnt)-β-catenin signaling pathway has been implicated in resistance mechanisms. Overexpression of Wnt family member 3 (Wnt3) can elevate β-catenin levels, promoting cell growth and invasiveness. However, the precise role of Wnt/β-catenin signaling in mediating ADC resistance remains incompletely understood and warrants further investigation^[48]^.

## GROWTH FACTOR SIGNALING PATHWAYS

Alterations in growth factor signaling pathways can also modulate cellular sensitivity to cytotoxic agents and ADCs. Neuregulin-1 (NRG1), a ligand that activates the HER3 receptor, is frequently upregulated - along with HER3 itself - in drug-resistant cell lines. This co-upregulation contributes to resistance against HER2-targeted therapies. Notably, inhibition of the NRG1/HER3 axis has been shown to enhance tumor suppression and delay disease recurrence^[49]^. Furthermore, loss of tuberous sclerosis complex 1 and 2 (TSC1 and TSC2), which serve as negative regulators of the mTOR complex 1 (mTORC1), confers greater resistance to T-DM1 compared with wild-type cells^[50]^. Encouragingly, mechanistic target of rapamycin (mTOR) inhibitors may help overcome ADC resistance by promoting lysosomal processing - a phenomenon observed with combinations of everolimus and T-DM1^[51]^.

## ROR1, HIPPO/YAP PATHWAY, AND SRC FAMILY KINASES

Another resistance mechanism involves the receptor tyrosine kinase-like orphan receptor 1 (ROR1). Overexpression of ROR1 can induce T-DM1 resistance even under conditions of adequate drug exposure. Simultaneously, activation of the Hippo/yes-associated protein (YAP) signaling pathway enhances tumor cell stemness and self-renewal capacity, further promoting therapeutic resistance^[52]^. In addition, phosphorylation of Src family kinases (SFKs) is markedly elevated in resistant cell lines. *YES1* gene amplification results in overexpression of Yes kinase, thereby contributing to cross-resistance against HER2-targeted therapies^[53]^.

## DNA REPAIR PATHWAYS

The DNA repair pathway also plays a critical role in mediating resistance to HER2-targeted ADCs^[54]^. For example, the structure-specific endonuclease subunit SLX4 (*SLX4*) gene, which is essential for DNA damage repair, has been shown to influence ADC responsiveness - tumor cells lacking *SLX4* expression exhibit resistance to T-DXd^[55]^. Moreover, Polo-like kinase 1 (PLK1), a key regulator of mitosis, is upregulated in T-DM1-resistant cells. Inhibition of PLK1 can activate the caspase-3/cyclin-dependent kinase 1 (CDK1)/B-cell lymphoma 2 (Bcl-2) signaling cascade, induce DNA damage, and potentially restore the antitumor activity of ADCs^[56]^.

## CELL CYCLE AND APOPTOSIS PATHWAYS

Resistance can also emerge through alterations in cell cycle regulation and apoptotic signaling. Cyclin B1, a key regulator of the G2/M transition of the cell cycle (G_2_-M) phase transition, accumulates in T-DM1-sensitive cells but not in resistant counterparts. Suppression of cyclin B1 upregulation can partially restore T-DM1 sensitivity, suggesting that cyclin B1 expression levels may serve as a potential biomarker of T-DM1 efficacy^[57]^. In addition, dysregulation of apoptotic pathways can modulate cellular responsiveness to cytotoxic agents. Expression of the B-cell lymphoma-extra large (*BCL-XL*) gene has been shown to correlate with the intrinsic sensitivity of non-Hodgkin lymphoma cells to ADCs^[58]^. Furthermore, inhibition of Bcl-2 family proteins has been found to potentiate ADC activity, offering a promising therapeutic strategy to overcome resistance^[59]^.

In summary, multiple signaling pathways - including PI3K, Wnt/β-catenin, growth factor signaling, Hippo/YAP, DNA repair mechanisms, and apoptotic regulators - contribute to the development of ADC resistance. These pathways may function independently or interact synergistically, highlighting the multifactorial and interconnected nature of resistance mechanisms. A deeper understanding of these molecular alterations will be crucial for guiding the design of next-generation therapies and identifying predictive biomarkers to overcome resistance, ultimately enhancing the efficacy and durability of ADC-based treatments.

## STRATEGIES TO OVERCOME RESISTANCE TO ADCS

In response to the multifaceted mechanisms driving ADC resistance, a variety of rational strategies have been proposed. These include optimizing antigen or target selection, engineering more efficient linker-payload systems, modulating the immune response, and designing combination regimens that synergistically overcome resistance barriers.

### Target optimization

To optimize therapeutic efficacy, novel bispecific ADCs have been developed, primarily following two design strategies. One approach targets two distinct epitopes on the same antigen - commonly referred to as biparatopic ADCs, while the other simultaneously targets two different antigens. The former enhances binding avidity and minimizes antigen escape, whereas the latter improves antigen specificity. Both designs strengthen ADC-antigen interactions and collectively enhance therapeutic potency^[60]^.

Studies have shown that bispecific ADCs targeting programmed death-ligand 1 (PD-L1) and B7 homolog 3 (B7-H3) not only deliver cytotoxic payloads to tumor cells with high selectivity but also activate the immune system through multiple mechanisms, including immune checkpoint inhibition and immunogenic cell death, thereby achieving more potent immunotherapeutic effects^[9]^. Recent research further demonstrates that antibody-T moiety-exatecan conjugates targeting HER2, HER3, and TROP-2 can effectively overcome both intrinsic and acquired resistance observed with equivalent DXd or SN-38-based ADCs, particularly in tumors characterized by low antigen expression, high tumor burden, or multidrug resistance. When SN-38 is used in ADCs such as sacituzumab govitecan and labetuzumab govitecan, modification of the antibody with boronate linkers can restrict cytotoxic activity specifically to tumor cells^[11]^. These next-generation ADCs exhibit superior therapeutic indices, enhanced molecular stability, and improved intratumoral pharmacodynamics, collectively advancing treatment outcomes^[61]^.

Recent investigations have also focused on lipid-based nanoparticle-mediated targeted drug delivery systems for anticancer therapy. In this approach, lipid nanoparticles (LNPs) are synthetically engineered with a lipid core conjugated to an albumin stealth coating and functionalized with targeting antibodies via thiol chemistry. Antibody conjugation markedly enhances cell-specific targeting, while LNPs are efficiently internalized through the endocytic pathway. Combining ease of synthesis with high serum stability, this platform has demonstrated strong potential to improve the drug delivery efficiency of ADCs, thereby increasing therapeutic efficacy while reducing systemic toxicity^[62]^. Moreover, antibody-functionalized LNPs can selectively bind to receptors overexpressed on angiogenic endothelial or tumor cells, resulting in improved targeting precision and binding affinity^[63]^.

Collectively, these advances underscore the promise of lipid-based nanocarriers as next-generation delivery platforms for ADCs, offering enhanced antitumor potency with minimized off-target effects and paving the way for more effective and safer targeted cancer therapies.

### Payload modifications

Next-generation ADC payloads are being designed to overcome efflux-mediated resistance by employing novel cytotoxic agents that are poor substrates for drug efflux pumps. Replacing conventional payloads with such agents represents a key strategy to circumvent ADC resistance. This approach has demonstrated potent antitumor activity in acute myeloid leukemia models, offering a promising avenue for restoring ADC efficacy^[59]^. Given the established role of ABC transporters in drug efflux, inhibitors targeting these transporters are being explored in combination with ADCs to mitigate resistance. For instance, ATP-binding cassette sub-family G member 2 (ABCG2) [breast cancer resistance protein (BCRP1)] was found to be significantly upregulated in NCI-N87 human gastric carcinoma cell line (N87) T-DXd-resistant cells compared with parental lines, and co-treatment with a potent and selective ABCG2 (BCRP) inhibitor (Ko143) or a small-molecule inhibitor of ABCG2 (BCRP) (KS176) successfully restored T-DXd sensitivity, providing early preclinical evidence for combination therapy^[64]^. Similarly, combining anti-Nectin-4 ADCs with multidrug resistance protein 1 (MDR1)/P-glycoprotein inhibitors reversed resistance *in vivo* while maintaining tolerable toxicity levels in breast cancer models^[32]^.

Other combination strategies are also under investigation^[65]^. In a HER2-transgenic breast cancer mouse model, co-administration of T-DXd with CD47 checkpoint blockade significantly enhanced antitumor immune responses and induced durable CD8^+^ T cell memory following treatment cessation^[20]^. In HER2-directed ADC-resistant breast cancer cell lines, loss of erb-b2 receptor tyrosine kinase 2 (*ERBB2*) gene amplification, driven by DNA damage and epigenetic mechanisms, further underscores the importance of the DNA repair pathway as both a canonical resistance mechanism and a promising therapeutic target to improve T-DXd efficacy^[54]^.

Recent efforts have also focused on site-selective and site-specific conjugation chemistries to construct homogeneous multi-payload ADCs. These engineered constructs feature two or more distinct cytotoxic payloads conjugated to defined locations on the antibody in a spatially controlled manner. Such ADCs can be categorized based on whether functionalization occurs at a single or multiple antibody sites^[66]^. Incorporating different types of payloads allows ADCs to bypass efflux mechanisms, making payload diversification a promising strategy to enhance therapeutic potency. For example, SKB-264 (also known as MK-2870), a TROP-2-targeting ADC, demonstrated improved efficacy through substitution of its TOP1 inhibitor payload with a belotecan derivative^[67]^. Likewise, zanidatamab zovodotin (ZW49), a bispecific antibody targeting two non-overlapping epitopes of HER2, achieved an objective response rate of 31% in patients with solid tumors^[68]^.

The optimal design strategy for these advanced ADCs depends on factors such as payload type, number of distinct payloads, DAR, and the *in vivo* properties of the antibody^[66]^. Combining complementary therapeutic mechanisms may yield synergistic effects. For instance, pairing ADCs with cytotoxic agents that act through distinct mechanisms, or exploiting synthetic lethality - such as combining ADCs with poly(ADP-ribose) polymerase (PARP) inhibitors - shows considerable therapeutic potential^[69]^. Nonetheless, toxicity remains a major concern in combination therapy. Reports on several anti-HER2 dual-drug ADCs indicate that, while delivering two small-molecule drugs with distinct mechanisms via a single antibody enhances cytotoxic potency, such designs must balance efficacy with tolerability^[70]^.

In addition, innovative strategies such as tumor-targeted nano-photothermal agents based on anti-HER2 monoclonal antibodies, utilizing supramolecular self-assembly to synergize with ADCs, have shown encouraging preclinical results^[71]^. Sequential administration of different ADCs targeting distinct antigens may also reduce the risk of cross-resistance, further broadening therapeutic options^[4]^. Emerging research is also exploring alternative payloads, including peptides and radionuclides, as part of the ongoing effort to optimize ADC payload selection^[27]^.

Collectively, these advances underscore the importance of innovative payload design, combination strategies, and novel delivery systems in overcoming resistance while preserving safety. However, the scarcity of comprehensive *in vivo* biological data highlights the urgent need for future studies to identify ADC combinations that deliver synergistic efficacy with manageable toxicity profiles.

### Linker innovations

The optimization of linker stability is intricately linked to target selection, payload properties, and the tumor microenvironment, all of which collectively determine the extent of extracellular payload release and the overall therapeutic performance of an ADC. An effective linker must maintain plasma stability, securely tethering the cytotoxic payload to the antibody until the ADC is internalized by cancer cells. By precisely controlling the timing and site of payload release, such linkers play a pivotal role in maximizing therapeutic efficacy while minimizing systemic toxicity^[6]^. The development of both stable and cleavable linkers has markedly improved payload delivery efficiency. For instance, replacing an auristatin-based ADC with a cleavable linker has been shown to overcome multidrug resistance-associated protein 1 (MRP1)-mediated resistance to T-DM1^[72]^. Ultimately, the selection of optimal linkers for conjugating nucleotides to antibodies, coupled with identification of ideal antibody conjugation sites, will be key to advancing next-generation biologics-based ADCs.

Exploiting the acidic tumor microenvironment represents another promising strategy for linker optimization. pH-sensitive linkers can be designed to exploit the differential pH between tumor tissues and normal cells. Preclinical studies have demonstrated that pertuzumab exhibits pronounced pH sensitivity, with rapid dissociation from HER2 under acidic conditions. Building on these observations, an experimental recombinant pertuzumab-based ADC was developed and has shown enhanced cytotoxicity in early studies^[73]^.

Moreover, rational drug-linker engineering offers additional therapeutic opportunities. Upon lysosomal degradation, the released cytotoxic payload may no longer act as a substrate for multidrug efflux pumps, thereby mitigating one of the major mechanisms of resistance. Current research emphasizes optimizing the attachment site and chemistry of the drug-linker interface to achieve efficient internalization and controlled payload liberation. Labile or cleavable linkers enable ADCs to enter cells via antigen-mediated endocytosis, effectively bypassing small-molecule efflux pumps located on the plasma membrane^[74]^. In parallel, the strategic incorporation of non-cleavable linkers, in combination with such design strategies, can further enhance therapeutic efficacy by limiting efflux-mediated drug loss^[75]^. Because hydrophobic compounds are more efficiently transported by efflux pumps than hydrophilic ones, modulating linker hydrophilicity presents another viable approach. By increasing linker hydrophilicity, researchers can reduce susceptibility to efflux, improving intracellular drug retention and enhancing the overall therapeutic index^[76]^.

### Immune modulation

Enhancing antitumor immunity represents a promising strategy to overcome drug resistance. By combining ADCs with agents that activate antitumor immune responses - such as immune checkpoint inhibitors (ICIs) - it is possible to achieve synergistic therapeutic effects. ADCs can stimulate antitumor immunity by activating antigen-presenting cells (APCs) and inducing intrinsic immunogenic tumor cell death, thereby amplifying immune-mediated tumor eradication. Several clinical trials are currently investigating ADC-ICI combination therapies, reflecting growing interest in this dual-modality approach^[77,78]^.

In parallel, advances in antibody engineering aim to enhance Fc-mediated immune functions. Glycoengineering and amino acid substitutions have been shown to improve ADCC by increasing Fc gamma (Fcγ) receptor binding affinity^[36]^. Although these strategies have shown promise, their clinical applicability in ADC design remains under active evaluation.

In recent years, a novel class of agents known as immune-stimulating antibody conjugates (ISACs) has emerged. These next-generation therapeutics integrate the targeting precision of monoclonal antibodies with the immunomodulatory potency of small-molecule immune agonists, acting on both the innate and adaptive immune systems. By conjugating antibodies to immune-stimulatory payloads, ISACs can enhance immune activation within the tumor microenvironment. For example, combining ADCs with immune checkpoint blockade has been shown to increase CD8^+^ effector T-cell infiltration, alleviate immune suppression, and improve overall therapeutic outcomes^[79]^.

The development of immunomodulatory ADCs (iADCs) represents another frontier in this field. One example is a trastuzumab biosimilar conjugated to a Toll-like receptor 7/8 (TLR7/8) agonist, designed to activate APCs and potentially overcome therapeutic resistance associated with tumor heterogeneity^[80]^. The antibody component confers tumor-specific targeting, ensuring localized delivery of immune agonists while minimizing systemic exposure and off-target toxicity. This dual mechanism - combining immune activation with selective tumor targeting - not only enhances therapeutic efficacy but also mitigates the safety concerns often associated with traditional immune agonist therapies^[81]^.

Compared with conventional ADCs, immune-modulating ADCs expand the therapeutic scope by targeting a broader range of antigens and achieving efficacy at lower payload doses. While traditional ADCs rely primarily on antigen-mediated endocytosis to deliver cytotoxic payloads, these next-generation constructs instead leverage Fcγ receptor-mediated endocytosis on immune cells, shifting the therapeutic paradigm from direct tumor cell killing to immune system engagement and activation^[81]^.

A notable example is OMTX705, a novel humanized anti-fibroblast activation protein (FAP) antibody conjugated to cytolysin TAM470, which has demonstrated significant tumor regression and delayed progression in immunocompetent models resistant to programmed cell death protein 1 (PD-1) inhibition. These effects appear to be mediated by CD8^+^ T cell-dependent immunomodulatory mechanisms, underscoring the potential of this new therapeutic class to overcome resistance and broaden the efficacy of immuno-oncology strategies^[82]^.

## FUTURE DIRECTIONS

**Figure 3:**
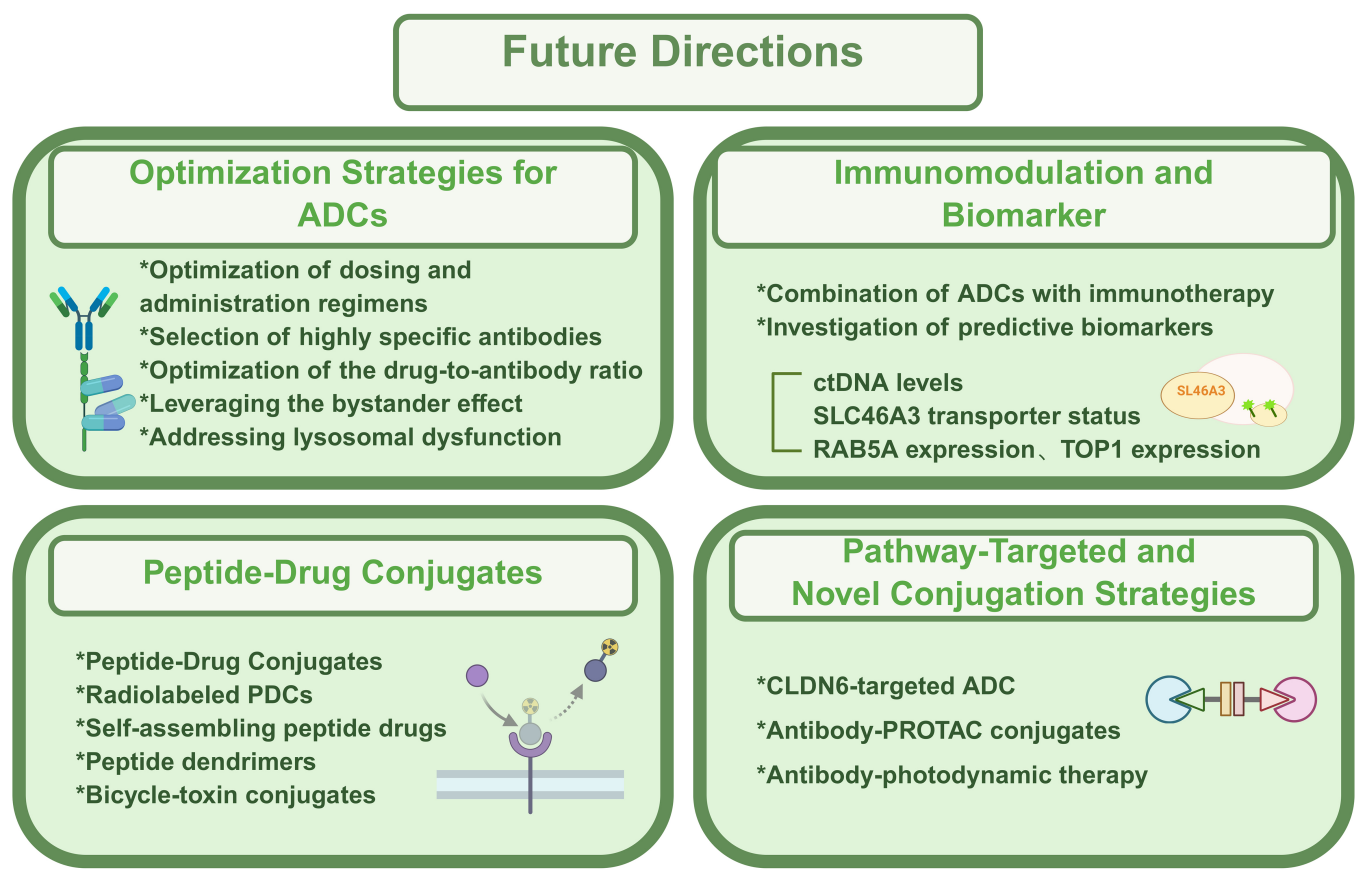
Main future directions of ADC. This summary outlines recent advances in targeted conjugate therapies, including optimization of ADC design (dose, specificity, DAR, and resistance mechanisms), integration with immunotherapy and biomarker discovery, and the emergence of PDCs offering improved safety and tumor selectivity. Novel strategies such as CLDN6-targeted ADCs, Ab-PROTACs, and antibody-photodynamic therapies further expand precision oncology’s potential. Created in BioRender. Hao, Y. (2025) https://BioRender.com/8mu5axq. ADC: Antibody-drug conjugate; DAR: drug-to-antibody ratio; PDCs: peptide-drug conjugates; CLDN6: Claudin-6; Ab-PROTACs: antibody-proteolysis-targeting chimera conjugates; ctDNA: circulating tumor DNA; SLC46A3: solute carrier family 46 member A3; RAB5A: Ras-related protein Rab-5A; TOP1: topoisomerase I; PROTAC: proteolysis-targeting chimera.

Amid the rapid pace of innovation in oncology drug development, ADCs occupy a unique position at the crossroads of opportunity and challenge^[83]^. This section outlines emerging therapeutic directions and conceptual strategies that may shape the next generation of ADCs. The details were illustrated in Figure 3.

Given their structural complexity, optimization of dosing and administration regimens is critical to maximize efficacy while minimizing systemic toxicity. Selecting antibodies with high specificity and affinity - while reducing immunogenicity - can limit off-target effects on normal tissues. Because dose-limiting toxicities often arise in cells lacking target antigen expression, further research is needed to mitigate off-target effects caused by premature drug release or nonspecific uptake^[84]^. Optimizing the DAR by fine-tuning the number of payloads per antibody is also essential for improving the therapeutic index. Moreover, leveraging the bystander effect, refining the physicochemical properties of ADC metabolites, and developing novel antigen-targeting strategies can enhance efficacy in tumors with heterogeneous target expression. For instance, cleavable disulfide linkers enable the release of neutral catabolites capable of diffusing across cell membranes, thereby eliminating adjacent cells with low antigen expression through a bystander mechanism. In addition, addressing lysosomal proteolytic dysfunction - a known resistance mechanism - through strategies that restore lysosomal activity may further improve ADC performance^[85]^.

Beyond payload and linker optimization, the integration of immunomodulatory agents into ADC platforms is expanding therapeutic potential. Emerging ADC-immunotherapy combinations show promise in enhancing antitumor immunity. Simultaneously, efforts are underway to identify predictive biomarkers that can inform patient selection and resistance monitoring. For example, biomarkers such as circulating tumor DNA (ctDNA) levels, SLC46A3 transporter status, and Ras-related protein Rab-5A (RAB5A) expression - an endocytic trafficking regulator from the RAS oncogene family - are under investigation. Elevated RAB5A expression has been correlated with increased responsiveness to T-DM1, suggesting that endocytic trafficking-related proteins may serve as predictive biomarkers for ADC efficacy^[86]^. Similarly, the expression of downstream molecular targets such as TOP1, which is highly expressed across several tumor types including breast and lung cancers, may provide additional insight into therapeutic outcomes^[87]^.

A complementary class of therapeutics, peptide-drug conjugates (PDCs), has demonstrated superior efficacy compared to peptides or small molecules alone. Akin to ADCs, PDCs exert their effects through multifactorial mechanisms but offer advantages such as reduced off-target toxicity and enhanced site-specific conjugation, translating into improved safety and broader therapeutic potential^[88]^. Radiolabeled PDCs have gained traction in theranostic applications. For instance, DOTA-(Tyr^3^)-octreotide (^68^Ga-DOTATOC) rapidly localizes to tumors, achieving approximately 80% uptake within 30 min and a target-to-nontarget ratio of 100:1 in central nervous system lesions compared to normal brain tissue^[89]^.

One notable example is Angiopep-2–paclitaxel conjugate (ANG1005), a next-generation PDC comprising three paclitaxel moieties covalently attached to Angiopep-2. Designed to traverse the BBB and blood-cerebrospinal fluid barrier via low-density lipoprotein receptor–related protein 1 (LRP1)-mediated transport, ANG1005 enables efficient intracellular drug delivery to malignant cells^[90]^. Clinical trials in heavily pretreated patients with advanced solid tumors, including those with brain metastases and taxane resistance, have demonstrated encouraging efficacy and tolerability, validating the clinical promise of PDCs^[90]^. Emerging PDC formats - such as bicycle-toxin conjugates, peptide dendrimer conjugates, and self-assembling peptide drug conjugates - are also advancing rapidly in preclinical and clinical development^[91]^. A particularly exciting example is a C-X-C chemokine receptor type 4 (CXCR4) antagonist peptide-docetaxel conjugate, which self-assembles into nanoparticles targeting CXCR4-overexpressing metastatic tumors. This agent has shown remarkable efficacy in suppressing bone and lung metastases in TNBC, highlighting the potential of PDC-based nanotechnologies to transform cancer therapy^[92]^.

Meanwhile, therapeutic strategies targeting specific signaling pathway alterations continue to evolve. The re-expression of Claudin-6 (CLDN6) in hepatocellular carcinoma (HCC) has drawn attention as a potential target. Mechanistic studies have shown that CLDN6 promotes tumor progression via the CLDN6/tight junction protein 2 (TJP2)/yes-associated protein 1 (YAP1) axis, activating the Hippo signaling pathway. In response, a novel CLDN6-targeted ADC, consisting of an anti-CLDN6 monoclonal antibody conjugated with DM1, has exhibited promising antitumor activity in preclinical HCC models^[93]^.

Another innovation involves antibody-PROTAC conjugates (Ab-PROTACs), which merge ADC technology with the proteolysis-targeting chimera (PROTAC) platform - a breakthrough in chemical biology^[94]^. PROTACs function by recruiting target proteins to E3 ubiquitin ligases, leading to ubiquitination and proteasomal degradation. Owing to their catalytic mode of action, PROTACs achieve efficient degradation at low doses. When conjugated to antibodies, Ab-PROTACs combine the targeted delivery of ADCs with the catalytic degradation capability of PROTACs, potentially overcoming limitations of both modalities and establishing a powerful new therapeutic paradigm^[81]^.

Recent innovations have also explored antibody-mediated photodynamic therapies, wherein photosensitive agents are conjugated to tumor-specific monoclonal antibodies. Upon laser activation, these constructs induce rapid and localized cancer cell death while sparing surrounding normal tissues. This approach offers dual advantages - enhancing therapeutic precision and minimizing systemic toxicity - and represents a promising direction for future oncologic research^[81]^.

Finally, the clinical translation of ADCs continues to accelerate. To date, more than 100 ADC candidates are in various stages of clinical development. Continued progress will depend on strategic trial design and the incorporation of adaptive clinical strategies that address resistance mechanisms in real time. Such dynamic approaches will be critical to broadening the clinical impact of ADCs and ensuring their sustained role in next-generation cancer therapeutics.

## CONCLUSION

The mechanisms underlying resistance to ADCs are complex and multifactorial, including downregulation of target antigen expression, altered endocytic trafficking, enhanced drug efflux or cellular tolerance to payloads, lysosomal dysfunction, and dysregulation of key signaling pathways. Given this intricate landscape, the development of combination therapies and next-generation ADCs has become a critical priority.

Building upon the foundations of early ADC designs, emerging platforms - such as PDCs, immunomodulatory conjugates, radiolabeled ADCs, and multimodal systems including light-activated therapeutics and Ab-PROTACs - represent promising strategies to overcome resistance and broaden therapeutic scope. In parallel with these innovations, efforts to mitigate ADC-associated toxicities, particularly interstitial lung disease, remain essential to improving patient safety and quality of life.

Looking ahead, continued advances in ADC engineering, payload chemistry, and biomarker-driven clinical applications are expected to further expand the therapeutic potential of these agents, ultimately ushering in a new era of precision-targeted cancer therapy.

## DECLARATIONS

### Copyright

© The Author(s) 2025.
